# Supplementary material for: A Meta-Analysis of Comparative Transcriptomic Data Reveals a Set of Key Genes Involved in the Tolerance to Abiotic Stresses in Rice
Source: Int J Mol Sci. 2019 Nov 12;20(22):5662. doi: 10.3390/ijms20225662 (PMC6888222; doi:10.3390/ijms20225662)
Supplement: Supplementary file 1 [file ijms-20-05662-s001.zip › Fig_S4.pdf]

K==4

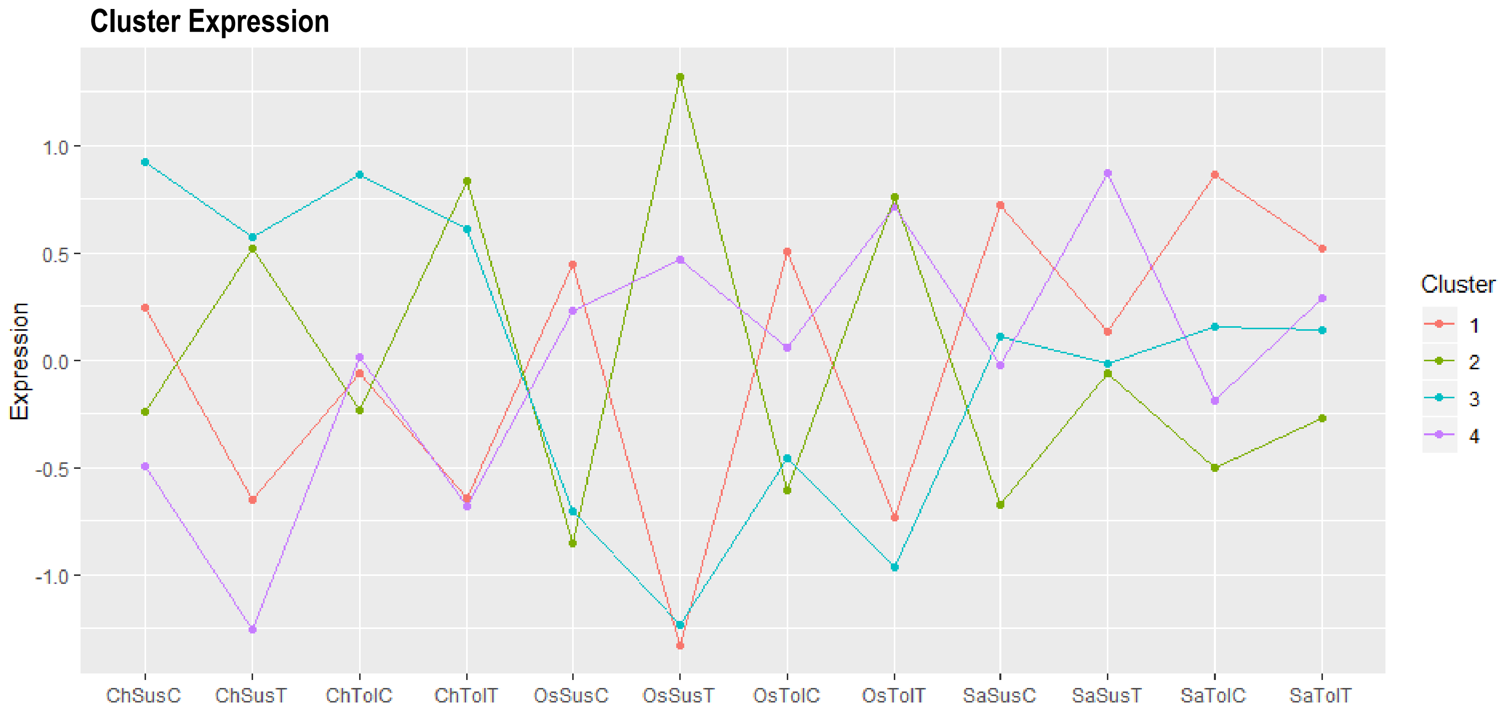


```
+ # Correlate the medoids to see how similar they are:
+   cor(kClustcentroids)
           1          2          3           4
1  1.00000000 -0.9507347  0.2802731  0.04631509
2 -0.95073468  1.0000000 -0.2392500 -0.04517640
3  0.28027314 -0.2392500  1.0000000 -0.65113785
4  0.04631509 -0.0451764 -0.6511379  1.00000000
```

Cluster 1 Expression

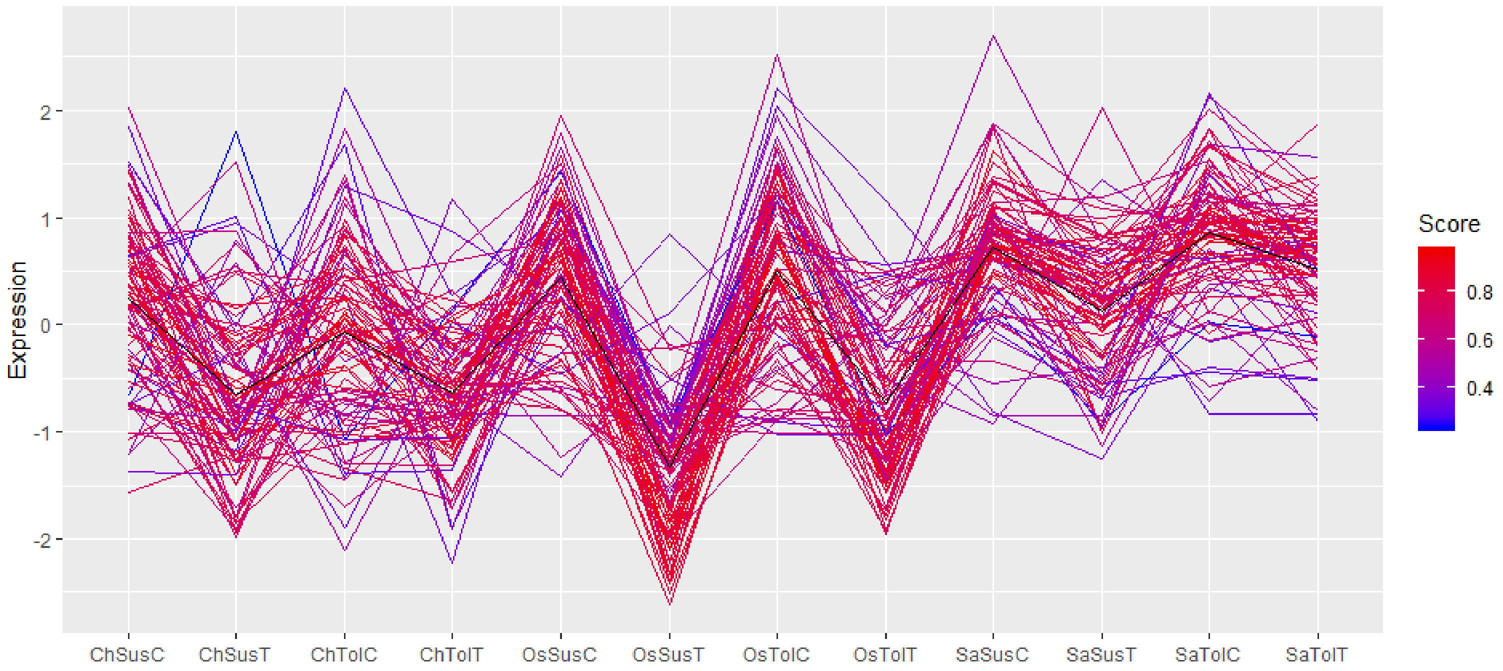

Cluster 2 Expression

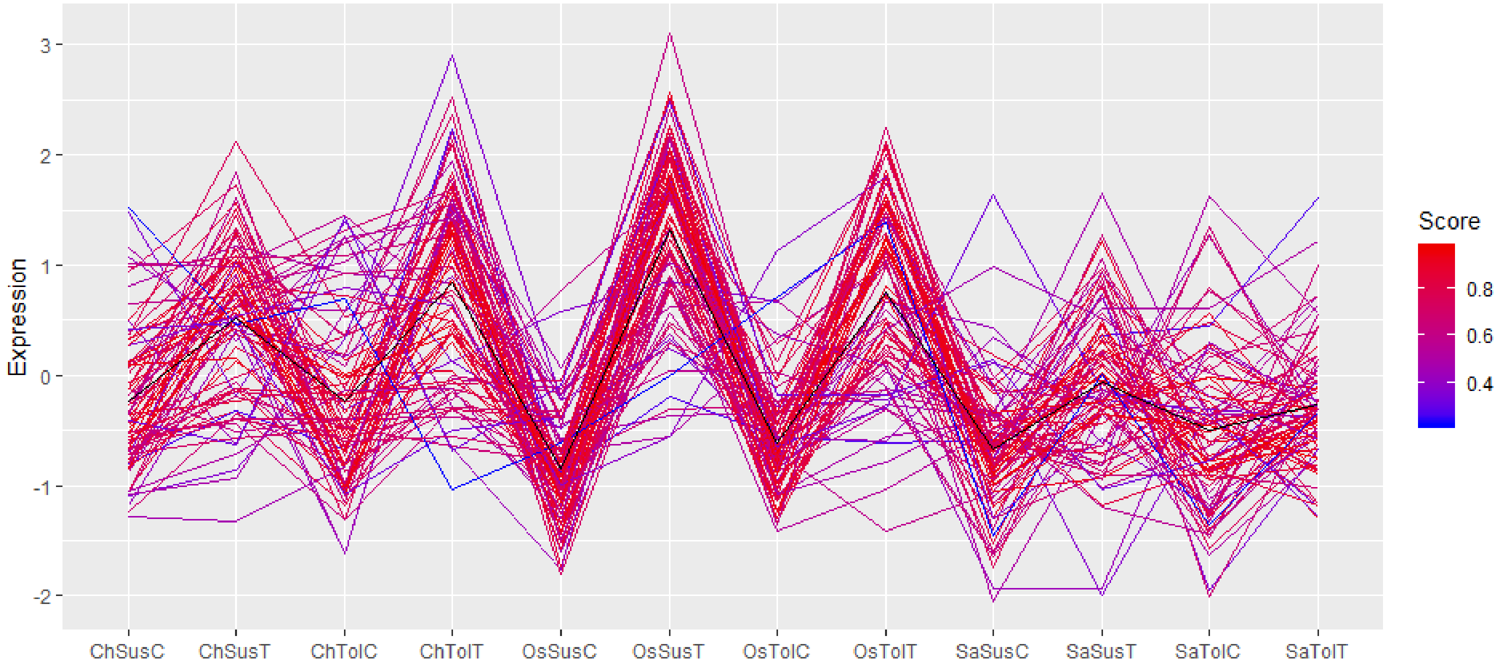

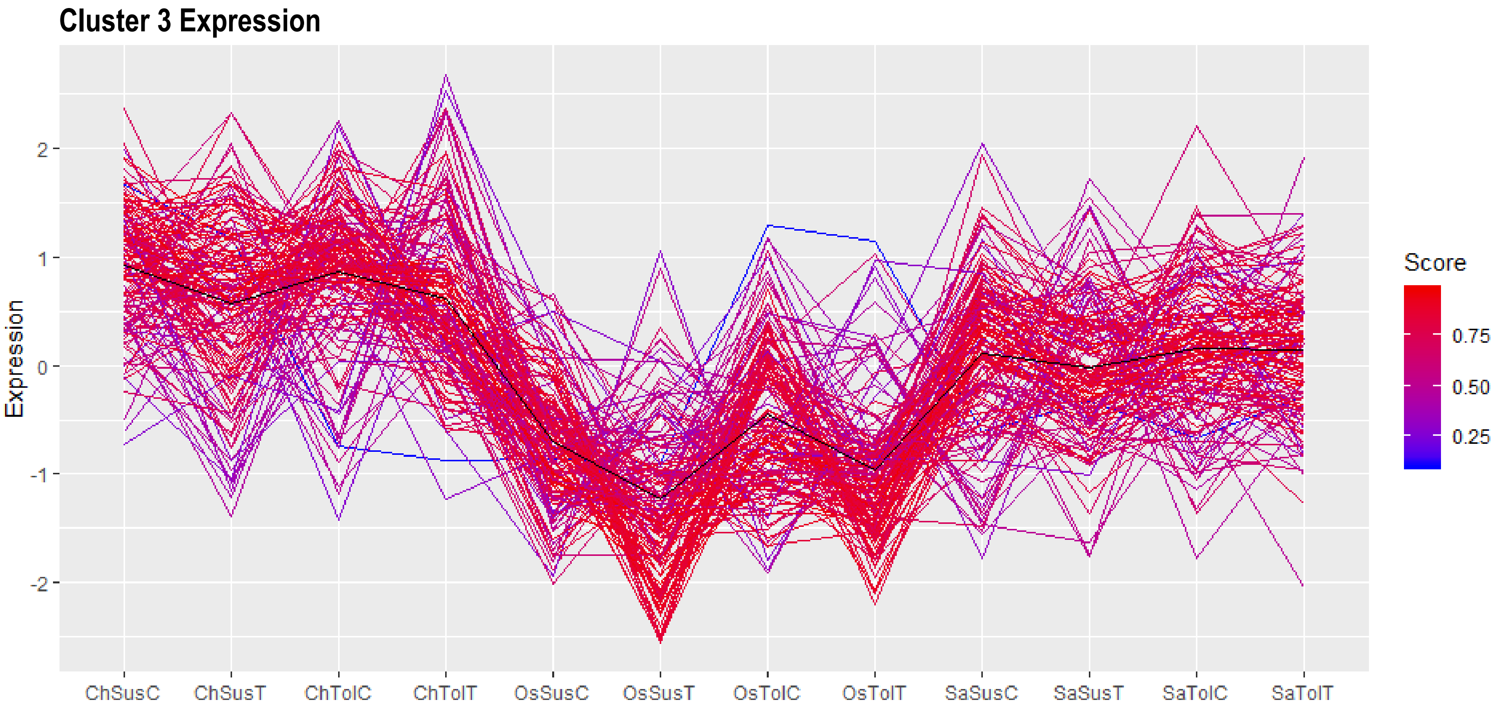
Cluster 3 Expression
Expression
2
1
0
-1
-2
ChSusC
ChSusT
ChTolC
ChTolT
OsSusC
OsSusT
OsTolC
OsTolT
SaSusC
SaSusT
SaTolC
SaTolT
Score
0.75
0.50
0.25

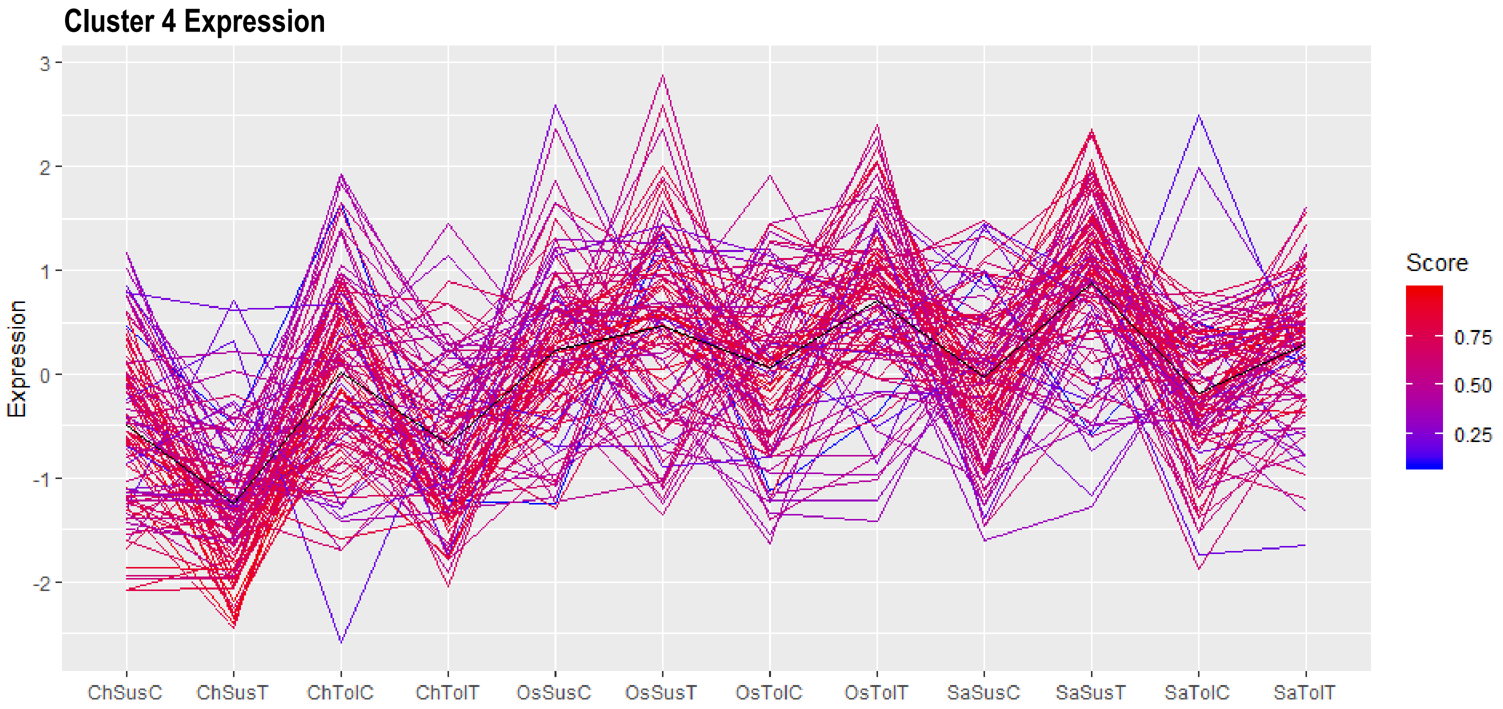
Cluster 4 Expression
Expression
3
2
1
0
-1
-2
ChSusC
ChSusT
ChTolC
ChTolT
OsSusC
OsSusT
OsTolC
OsTolT
SaSusC
SaSusT
SaTolC
SaTolT
Score
0.75
0.50
0.25
