# Supplementary material for: A Meta-Analysis of Comparative Transcriptomic Data Reveals a Set of Key Genes Involved in the Tolerance to Abiotic Stresses in Rice
Source: Int J Mol Sci. 2019 Nov 12;20(22):5662. doi: 10.3390/ijms20225662 (PMC6888222; doi:10.3390/ijms20225662)
Supplement: Supplementary file 1 [file ijms-20-05662-s001.zip › Supplementary Materials.pdf]

Supplementary Materials

Figure S1 Multi-dimensional scaling (MDS) plots for chilling, osmotic and salt stress RNA libraries generated with EdgeR. Plots represent the gene expression profiles differences between samples.

Figure S2 Scatter plots of mean $\log_2$(counts-per-million) versus $\log_2$(fold change). Transcriptional changes are presented for susceptible and tolerant cultivars in the three considered abiotic stresses. Significant DEGs ($FDR < 0.05$) for susceptible and tolerant cultivars are indicated with red and blue dots, respectively.

Figure S3 GO-enrichment analysis results of the 186 genes up-regulated in all the three stresses in both tolerant and susceptible cultivars.

Figure S4 Graphical representation of the four expression clusters identified with K-means clustering analysis.

Table S1 RNA libraries sequencing and mapping statistics. For each RNA library (genotypes in control and stress conditions, 3 biological replicates each) the number of raw reads, the number (and percentage) of reads after filtering out the adapters and low-quality sequences, and the number (and percentage) of reads mapped to *Oryza sativa* spp. *Japonica* (IRGSP1.0) genome are reported.

Table S2 Normalized reads counts for active genes in the investigated RNA libraries.

Table S3 EdgeR differential expression analysis results for all significant DEGs of stressed samples RNAs compared to control ones.

Table S4 Lists of up- and down-regulated genes ("Common Up-regulated" and "Common Down-regulated" sheets, respectively) in all the three stresses in both tolerant and susceptible cultivars. Chromosomal position and description according to RAP-DB annotation is reported for each gene.

Table S5 List of the genes putatively involved in the tolerant response of at least one of the three stresses. For each gene are reported: RAP-DB and MSU IDs, chromosomal location, description according to RAP-DB and, for the three stresses, the $\log_2$FC (LFC) values for susceptible and tolerant genotypes, their difference (if the gene is DE in both genotypes), and the classification of the genes in the six classes.

Table S6 List of the 420 genes putatively involved in the tolerant response of the three considered stresses. For each gene are reported: RAP-DB and MSU IDs, chromosomal location, description according to RAP-DB and, for the three stresses, the $\log_2$FC (LFC) values for susceptible and tolerant genotypes, the ΔLFC, if the gene is DE in both genotypes, the classification of the genes in the six classes, RPKM values.

Table S7 GO terms related to the 420 genes.

Table S8 Correlation matrix. Pearson correlation values (r) with $p < 0.005$ are reported.

Table S9 Cytoscape statistics of the GCN created fixing the Pearson's correlation value $|r| \geq 0.8$ (276 genes). The 112 highly co-expressed genes are highlighted in light orange.

Table S10 List of the genes belonging to the core of Gene Co-expression Network (subgroups A and B). For each gene the RAP ID, the gene name, the gene description, the subgroup, the cluster and the class of expression for each stress treatment are reported.

Table S11 List of the genes belonging to the *MYBs*-guided subnetwork. For each gene the RAP ID, the gene name, the gene description, the cluster, the class of expression for each stress treatment, and Pearson correlation values (r) with OsMYB55/61 and OsMYB61L are reported.

Table S12 Co-localization of genes listed in Table 4 within QTLs present in Q-TARO database. For each entry, position on rice genome and information about the QTL were reported.

Table S13 List of known QTLs for drought tolerance, cold tolerance and salt tolerance, as reported on Q-TARO database. The correspondence between stress-related regions (C01-C17, D01-D38, S01-S03) and QTLs is reported.
