# Supplementary figures and images for: A Meta-Analysis of Comparative Transcriptomic Data Reveals a Set of Key Genes Involved in the Tolerance to Abiotic Stresses in Rice
Source: Int J Mol Sci. 2019 Nov 12;20(22):5662. doi: 10.3390/ijms20225662 (PMC6888222; doi:10.3390/ijms20225662)

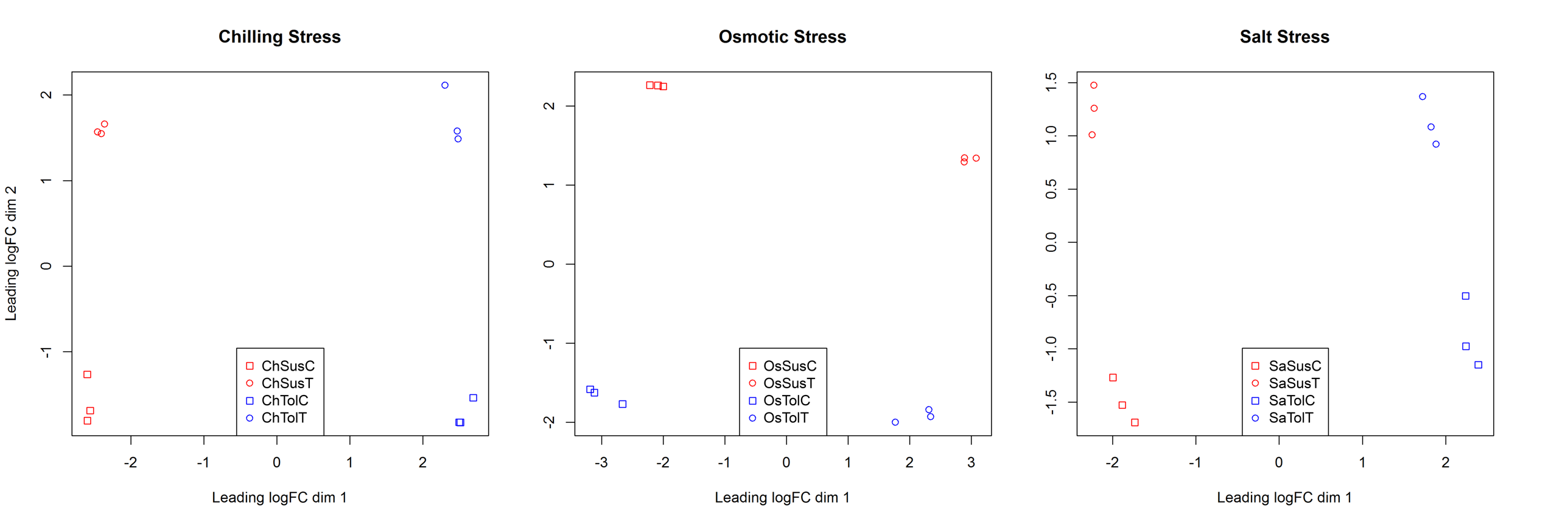

Supplement: Supplementary file 1 [file ijms-20-05662-s001.zip › Fig_S1.tif]

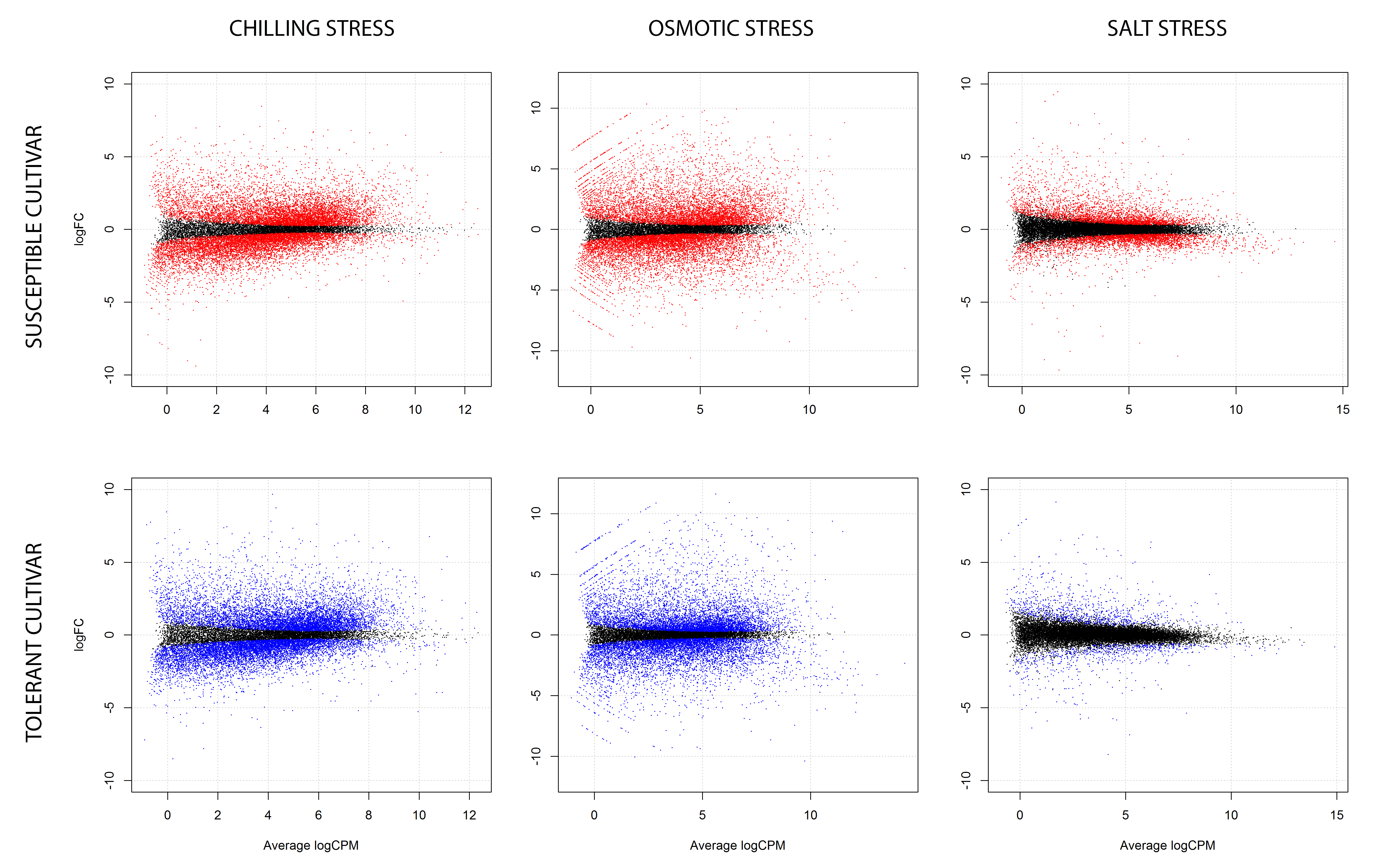

Supplement: Supplementary file 1 [file ijms-20-05662-s001.zip › Fig_S2.tif]

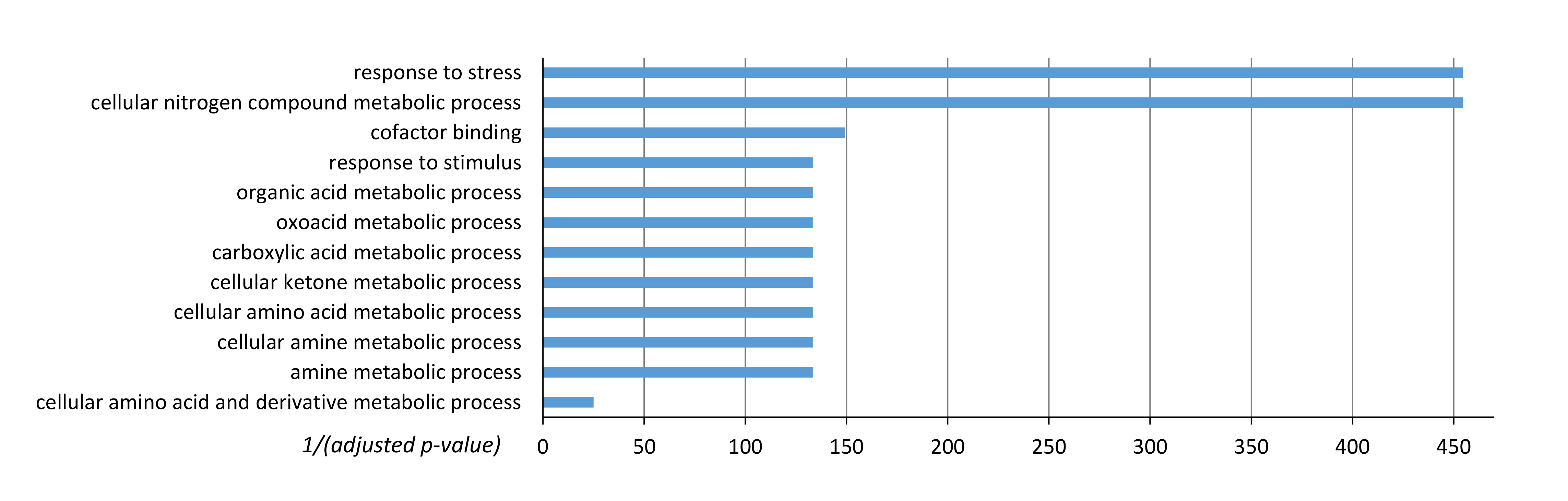

Supplement: Supplementary file 1 [file ijms-20-05662-s001.zip › Fig_S3.tif]

## SSE estimate

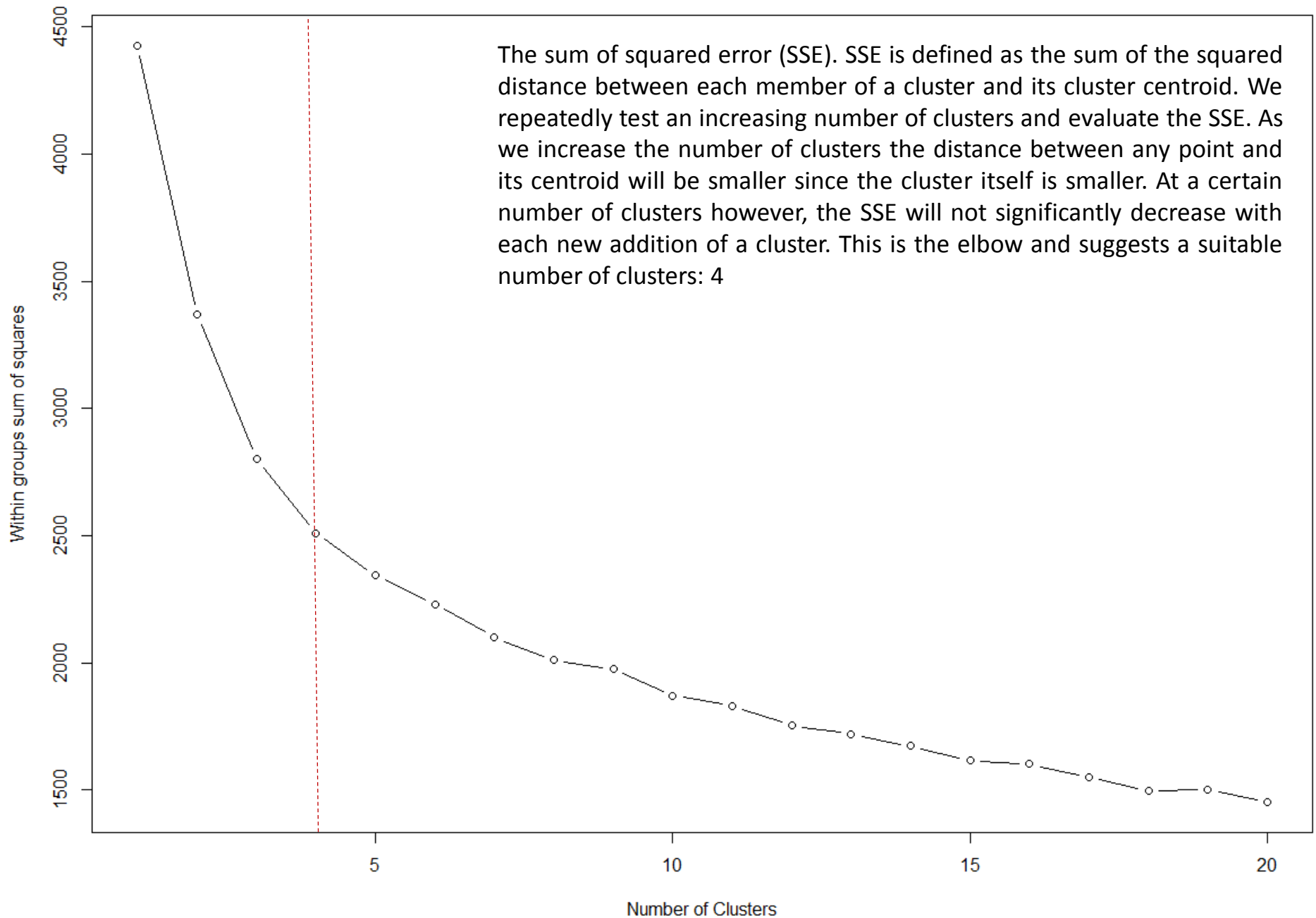

**K-means partitions comparison**

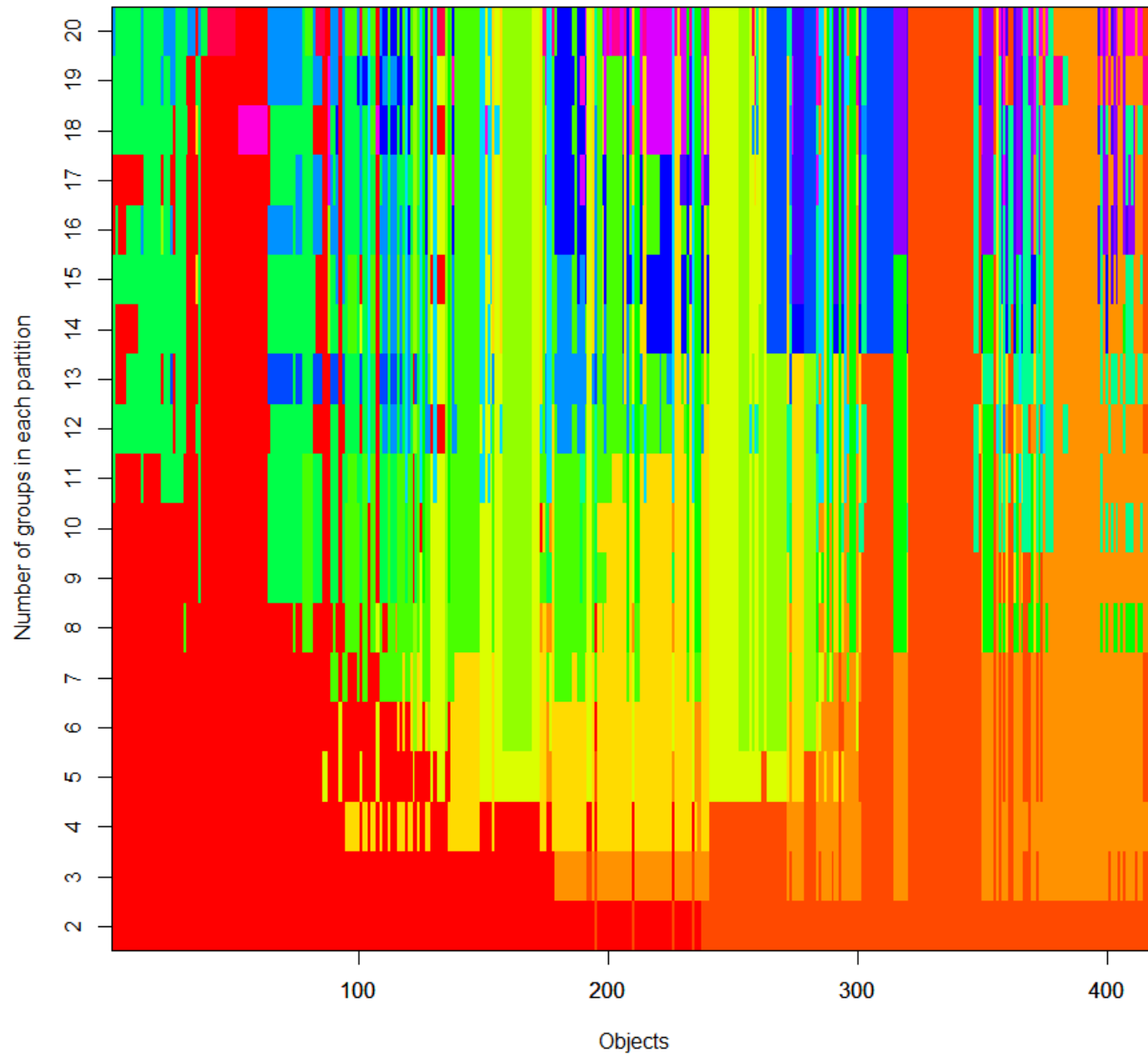

**calinski  
criterion**

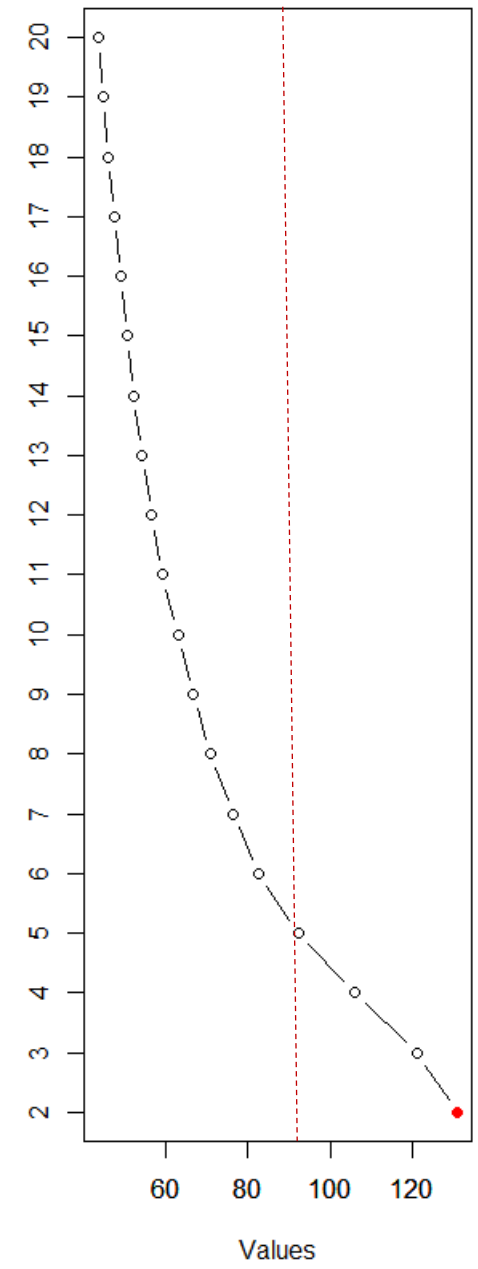

Supplement: Supplementary file 1 [file ijms-20-05662-s001.zip › Fig_S5.pdf]
